# RrgA is a pilus-associated adhesin in *Streptococcus pneumoniae*

**DOI:** 10.1111/j.1365-2958.2007.05908.x

**Published:** 2007-10

**Authors:** A L Nelson, J Ries, F Bagnoli, S Dahlberg, S Fälker, S Rounioja, J Tschöp, E Morfeldt, I Ferlenghi, M Hilleringmann, D W Holden, R Rappuoli, S Normark, M A Barocchi, B Henriques-Normark

**Affiliations:** 1 Swedish Institute for Infectious Disease Control and Department of Microbiology, Tumor and Cell Biology, Karolinska Institutet Stockholm, Sweden; 2 Novartis Vaccines Siena, Italy; 3 Centre for Molecular Microbiology and Infection, Imperial College London, UK

## Abstract

Adherence to host cells is important in microbial colonization of a mucosal surface, and *Streptococcus pneumoniae* adherence was significantly enhanced by expression of an extracellular pilus composed of three subunits, RrgA, RrgB and RrgC. We sought to determine which subunit(s) confers adherence. Bacteria deficient in RrgA are significantly less adherent than wild-type organisms, while overexpression of RrgA enhances adherence. Recombinant monomeric RrgA binds to respiratory cells, as does RrgC with less affinity, and pre-incubation of epithelial cells with RrgA reduces adherence of wild-type piliated pneumococci. Non-adherent RrgA-negative, RrgB- and RrgC-positive organisms produce pili, suggesting that pilus-mediated adherence is due to expression of RrgA, rather than the pilus backbone itself. In contrast, RrgA-positive strains with disrupted *rrgB* and *rrgC* genes exhibit wild-type adherence despite failure to produce pili by Western blot or immunoelectron microscopy. The density of bacteria colonizing the upper respiratory tract of mice inoculated with piliated RrgA-negative pneumococci was significantly less compared with wild-type; in contrast, non-piliated pneumococci expressing non-polymeric RrgA had similar numbers of bacteria in the nasopharynx as piliated wild-type bacteria. These data suggest that RrgA is central in pilus-mediated adherence and disease, even in the absence of polymeric pilus production.

## Introduction

Adherence to host cells is considered an important property of pathogenic microorganisms, bacterial, viral, fungal or otherwise (Mims, 1995). *Streptococcus pneumoniae* is a respiratory and invasive pathogen of humans and is responsible for an estimated 1–2 million deaths per year, primarily as a result of pneumonia (Mulholland, 1997). It is also a frequent colonizer of the upper respiratory tract of children, functioning as an ecological niche for the spread of these bacteria within the society. Thus, *S. pneumoniae*, also known as the pneumococcus, is a mucosal commensal, a mucosal pathogen and an invasive pathogen, and likely interacts with a variety of host cells. Indeed, the capacity of the pneumococcus to adhere to respiratory epithelial cells *in vitro*, for example, is thought to correlate with successful colonization and/or virulence *in vivo* (Tuomanen *et al*., 1995).

The pneumococcus is one of a number of Gram-positive organisms recently discovered to express a novel class of pili, distinct from known Gram-negative pili in the genetics, biochemistry and structure (Ton-That and Schneewind, 2004; Scott and Zahner, 2006; Telford *et al*., 2006). Pilus expression has been shown to enhance adherence to host cells in three streptococcal species, *S. pneumoniae* (Hemsley *et al*., 2003; Barocchi *et al*., 2006), *S. pyogenes* (Abbot *et al*., 2007) and *S. agalactiae* (Dramsi *et al*., 2006; Maisey *et al*., 2007), among others (Mandlik *et al*., 2007). Gram-positive pili also contribute to adherence to components of the extracellular matrix (Clark *et al*., 1984), to mucosal colonization in animal models (Barocchi *et al*., 2006), and to disease pathogenicity in animal models (Hava and Camilli, 2002; Barocchi *et al*., 2006; Lizano *et al*., 2007; Nallapareddy *et al*., 2006). In addition, antibodies directed against these structures are protective in animal models of disease in *S. agalactiae* (Maione *et al*., 2005), *S. pyogenes* (Mora *et al*., 2005) and *S. pneumoniae* (Gianfaldoni *et al*., 2007), and antibodies against pilus protein are present in the serum of patients suffering from enterococcal disease (Nallapareddy *et al*., 2006), suggesting that these structures are expressed on the Gram-positive bacterial surface during invasive disease.

All Gram-positive pili characterized to date are found on genetic loci encoding multiple structural subunits and one or more associated enzymes, homologues of sortase (Scott and Zahner, 2006; Telford *et al*., 2006). Sortase itself is nearly ubiquitous among Gram-positive bacteria, in which it is a ‘house-keeping’ enzyme that catalyses the covalent attachment of proteins containing C-terminal ‘sorting’ LPXTG-like motifs to the cell wall (Marraffini *et al*., 2006). In all cases studied thus far, pilus-associated sortases have been found to be important for the expression of mature extracellular pili polymers (Ton-That and Schneewind, 2003; Ton-That *et al*., 2004; Mora *et al*., 2005; Dramsi *et al*., 2006; Gaspar and Ton-That, 2006; Rosini *et al*., 2006), presumably by cross-linking individual pilus subunits to one another (Scott and Zahner, 2006). It is likely that the general sortase is responsible for addition of mature polymeric pili to the cell wall (Ton-That and Schneewind, 2003; Scott and Zahner, 2006). Genetic loci encoding Gram-positive pili are considered homologous to one another, although the numbers of subunits composing the pili vary, and the numbers of sortase enzymes participating in polymerization and attachment to cell surface also differ (Scott and Zahner, 2006; Telford *et al*., 2006). The biology of pilus assembly may be complex in cases where two (Dramsi *et al*., 2006; Gaspar and Ton-That, 2006; Rosini *et al*., 2006) or three (LeMieux *et al*., 2006) sortases have been identified in a given locus.

In the case of *S. pneumoniae* and other piliated Gram-positive organisms, it has been shown that assembly of the pilus is dependent upon expression of the major structural subunit of the pilus (Ton-That and Schneewind, 2003; Mora *et al*., 2005; Dramsi *et al*., 2006; LeMieux *et al*., 2006). The major structural subunit of the pneumococcal pilus is RrgB, the *rlrA*-regulated gene B (Hava and Camilli, 2002), named for the pilus-associated positive regulator, *rlrA* (Hava *et al*., 2003). In most cases of Gram-positive pili, in addition to the major pilin, there are two ancillary subunits (Scott and Zahner, 2006; Telford *et al*., 2006), called RrgA and RrgC in *S. pneumoniae* (Hava and Camilli, 2002). Western blot analysis of multiple Gram-positive pili demonstrates that the ancillary subunits are incorporated into the pilus (Ton-That and Schneewind, 2003; Mora *et al*., 2005; Barocchi *et al*., 2006; Dramsi *et al*., 2006; LeMieux *et al*., 2006; Rosini *et al*., 2006), presumably involving the so-called ‘E-box’ motif in the major pilin (Ton-That *et al*., 2004), although the specific molecular means by which this occurs are, as yet, unclear. Immunoelectron microscopic studies show that one ancillary subunit is incorporated at intervals along the length of the polymer (Ton-That and Schneewind, 2003; LeMieux *et al*., 2006) or at the base of the pilus (Barocchi *et al*., 2006), RrgA in the case of *S. pneumoniae*. The other ancillary subunit, pneumococcal RrgC, is preferentially found at the tip of the polymer (Ton-That and Schneewind, 2003; Barocchi *et al*., 2006).

Bioinformatic analysis of the genes encoding the three structural subunits of the pneumococcal pilus, *rrgA*, *rrgB* and *rrgC*, revealed the presence of adhesive MSCRAMM motifs (Patti *et al*., 1994) in all three (Hava and Camilli, 2002), and an adhesion-associated von Willebrand factor type A (vWA) (Colombatti and Bonaldo, 1991) (NCBI CDD accession number cd00198, Pfam accession number PF00092) domain and an adhesion-associated RGD motif (Ruoslahti and Pierschbacher, 1986) (ProSite accession number PS00016) in the *rrgA* gene product. It is not clear whether any of these motifs actually confers adhesive properties, but these observations suggest that any of the three subunits, and especially RrgA, may serve as adhesins. Studies in both *Streptococcus agalactiae* (Dramsi *et al*., 2006; Maisey *et al*., 2007) and *S. pyogenes* (Abbot *et al*., 2007) suggest that one of the ancillary subunits, the *rrgA* homologues, actually confers adherence to host cells, and recent work in *Corynebacterium diphtheriae* demonstrates that both ancillary subunits act as adhesins (Mandlik *et al*., 2007).

We sought to determine which subunit(s) of the pneumococcal pilus is required for adherence to host epithelial cells, and whether the pilus is actually necessary for this activity. Genetic data supported a single subunit as required for adherence, RrgA, even in the absence of polymer formation. Purified recombinant RrgA binds to epithelial cells, supporting a claim for RrgA as an adhesin. We then tested the importance of RrgA in a murine colonization model and found that RrgA, but not the other pilus structural subunits, are important for colonization of the upper respiratory tract in mice. These findings are especially surprising in light of the observation that RrgA can mediate adherence in multiple experimental models in the absence of the formation of a polymeric pilus. Understanding the role of the pilus itself, then, and the association of the pilus with adhesins that can apparently function independently, remains a major challenge of the field.

## Results

### Expression of the *rrgA* gene is required for pilus-mediated adherence

It has been shown that pneumococcal adherence to human lung epithelial cells is significantly enhanced by expression of the pilus (Hemsley *et al*., 2003; Barocchi *et al*., 2006). This experimental approach provides natural cognate host–pathogen interactions, a limitation of the mouse as an experimental host, which is not naturally colonized or infected by *S. pneumoniae*. Initial studies focused on mutants of a piliated, highly pathogenic serotype 4 strain, TIGR4, here called T4, originally isolated from a patient in Norway (Aaberge *et al*., 1995) that has been fully sequenced (Tettelin *et al*., 2001). T4 is highly pathogenic in mouse models (Aaberge *et al*., 1995; Sandgren *et al*., 2005). The pneumococcal pilus is known to contribute to T4 colonization and virulence in mouse models (Hava and Camilli, 2002; Barocchi *et al*., 2006), and pilus expression (Barocchi *et al*., 2006; LeMieux *et al*., 2006) and regulation (Hava *et al*., 2003; Hemsley *et al*., 2003) have been well documented in T4. To determine whether expression of RrgA, or the other two structural subunits, is required for pilus-mediated adherence, an insertion–deletion mutant was generated for *rrgA* (T4Δ*rrgA*), and a double-mutant of *rrgB* and *rrgC* (T4Δ*rrgBC*). The adherence of these two mutants to A549 human respiratory epithelial cells, in the absence of serum, was compared with wild-type T4 and a non-piliated mutant T4 strain in which all *rlrA*-regulated pilus islet genes other than *rlrA* had been deleted [T4Δ(*rrgA-srtD*)].

**Fig. 1:**
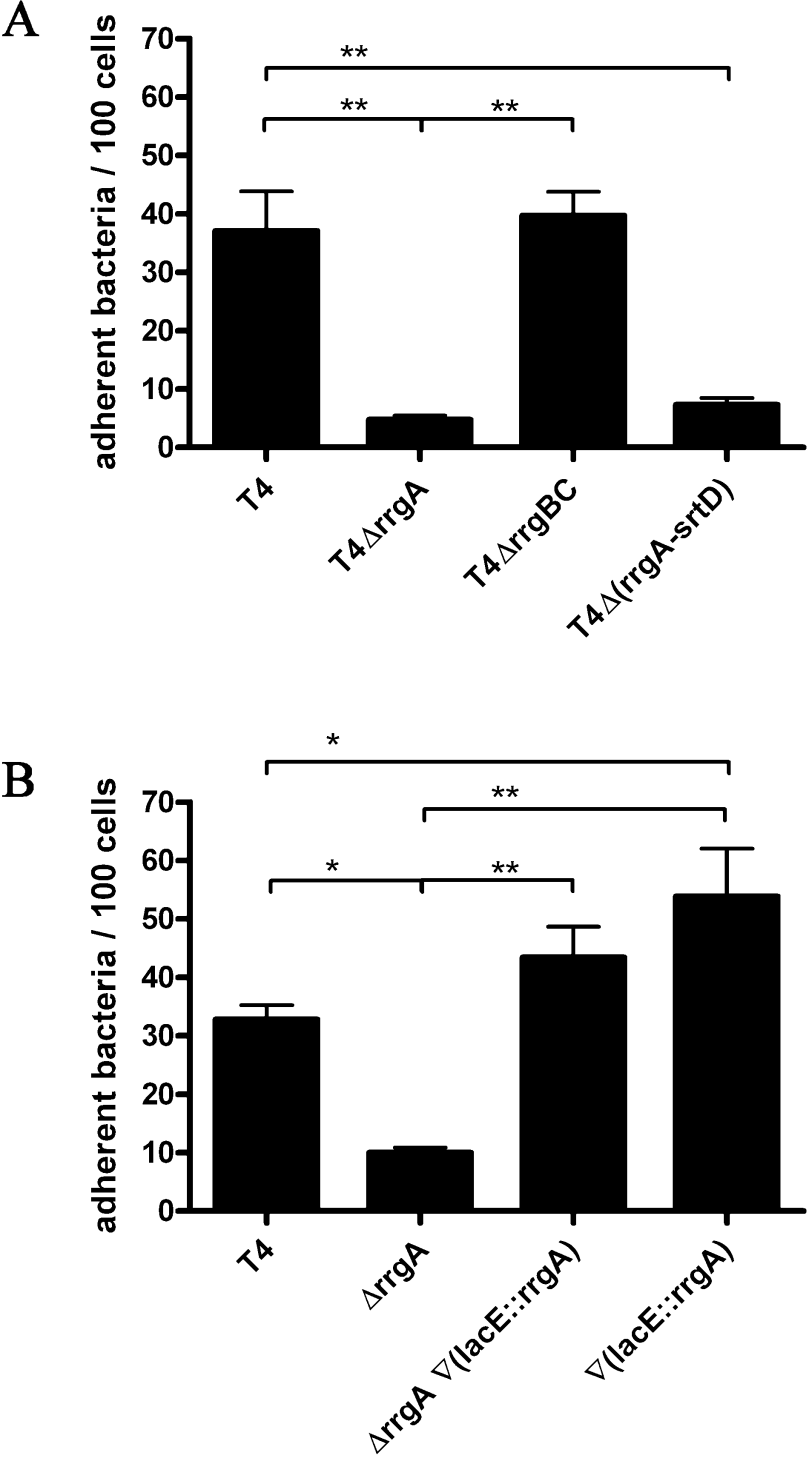
Expression of *rrgA* confers pilus-mediated adherence to human respiratory epithelial cells. A. *rrgA*, but not *rrgB* and *rrgC*, is necessary for pilus-mediated adherence to epithelial cells. Adherence of wild-type piliated TIGR4 (‘T4’) to A549 human respiratory epithelial cells was significantly greater than both non-piliated T4Δ(*rrgA-srtD*) compared with T4 deficient in the *rrgA* gene (‘T4Δ*rrgA’*). Adherence of T4 deficient in both *rrgB* and *rrgC* (‘T4Δ*rrgB*C’) was not notably different from wild-type organisms, but was significantly greater compared with T4Δ*rrgA*. Repeated-measure anova of data collected from three independent determinations indicates statistically significant differences within experimental conditions. *Post hoc* Bonferroni analyses identify specific significant differences: **P< 0.01. B. *rrgA* expression determines adherence to epithelial cells. T4 deficient in *rrgA* are significantly deficient in adherence (‘Δ*rrgA’*), compared with wild-type (T4), while *trans*-complementation restores wild-type adherence [‘Δ*rrgA*∇(*lacE*::*rrgA*’)]. Introduction of a second copy of *rrgA* inserted *in trans* in the *lacE* locus [‘∇(*lacE*::*rrgA*’] results in significant enhancement of adherence over wild-type levels. Statistical analyses were performed with repeated-measure anova of data collected from three independent determinations. *Post hoc* Bonferroni analyses identify specific significant differences: *P< 0.01 and **P< 0.001.

T4Δ*rrgA* was eightfold less adherent than wild-type T4, and therefore demonstrated adherence properties comparable to the non-piliated negative control strains, T4Δ(*rrgA-srtD*), which was fivefold less adherent than the wild-type parent (Fig. 1). T4Δ*rrgBC*, which expresses only the *rrgA* gene product, was found to be as adherent as the wild-type T4 strain, and was eightfold more adherent than T4Δ*rrgA*, and fivefold more adherent than T4Δ(*rrgA-srtD*) (Fig. 1). These data demonstrate that expression of RrgA, but not RrgB or RrgC, is required for pilus-mediated adherence to human respiratory epithelial cells. Further, expression of RrgA alone is sufficient to enhance adherence over non-piliated levels. In support of this model, it is notable that Δ*rrgA* organisms are out-competed by wild-type *S. pneumoniae* in a mouse model of pneumococcal pneumonia (Hava and Camilli, 2002).

To ensure *rrgA* was responsible for adherence, a second, intact copy of *rrgA* was inserted into the chromosome of T4Δ*rrgA* at a non-essential site, the *lacE* lactose utilization operon (Iyer *et al*., 2005), generating the strain T4Δ*rrgA*∇(*lacE::rrgA*). This strain expresses more RrgA-positive pili than wild-type pili, as assessed by Western analysis of cell wall-associated proteins (Fig S1). It is likely this is due to strong promoter activity in the *aad9* spectinomycin-resistance marker, and evidence of high levels of transgene expression with this system was noted in another study (A. Nelson *et al.*, in preparation). When tested in the described *in vitro* adherence, complementation of *rrgA in trans-*restored adherence to A549 monolayers, increasing adherence by approximately fourfold (Fig. 1B). In fact, the *trans* complemented strain appeared more adherent than wild-type (1.3-fold), commensurate with the high levels of RrgA expressed, although this difference was not statistically significant in *post hoc* analyses.

RrgA expression was further increased by transformation of wild-type T4 with the *lacE*::*rrgA trans*-complementation construct, resulting in a strain with two intact *rrgA* genes, T4∇(*lacE*::*rrgA*). This strain overproduces RrgA protein, as determined by Western analysis of cell wall-associated proteins (Fig S1). T4∇(*lacE*::*rrgA*) was found to be 1.6-fold more adherent to A549 respiratory epithelial cells than wild-type pneumococci, a significant difference (Fig. 1B). Thus, T4Δ*rrgA*, wild-type T4, T4Δ*rrgA*∇(*lacE*::*rrgA*), and T4∇(*lacE*::*rrgA*) express increasing amounts of RrgA-positive pili (Fig S1) and exhibit increasing levels of adherence. This correlation is genetic evidence of RrgA serving as a pilus-associated adhesin for *S. pneumoniae*.

### RrgA binds to A549 cell surface

**Fig. 2:**
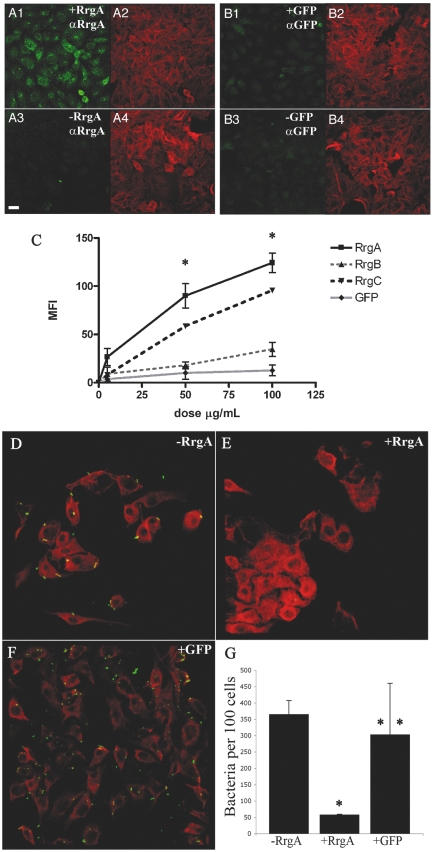
Recombinant RrgA and RrgC proteins bind to human respiratory epithelial cells. A–C. Recombinant RrgA binds directly to A549 respiratory epithelial cells. Cells were incubated with 100 μg ml^−1^ of purified RrgA (A1 and A2, ‘+RrgA’) or GFP (B1 and B2, ‘+GFP’) in DMEM culture medium, or medium alone (A3–4, ‘-RrgA’; B3–4, ‘-GFP’) for 2 h at 4°C. Cells were fixed and stained with anti-RrgA and anti-GFP antibodies (A1–4, ‘αRrgA’; B1–4, ‘αGFP’) and phalloidin, which served as a control to demonstrate the presence of cells (A2, A4, B2 and B4). Imaging was performed with a confocal microscope. Scale bar is 20 μm. C. RrgA and RrgC bind to A549 cells in a dose-dependent manner. A549 cells were mixed in suspension with either medium alone (0 μg ml^−1^) or three concentrations (5, 50 and 100 μg ml^−1^) of pilus subunits RrgA (squares with solid black lines), RrgB (triangles with dashed grey lines), RrgC (upside-down triangles with dashed black lines), or GFP protein (diamond with solid grey lines), incubated for 2 h at 4°C, stained with antisera specific to each protein, and detected with Alexa Fluor 488-conjugated secondaries. Cells were analysed with a FACS-Calibur flow cytometer, and the net mean fluorescence intensity for each population was calculated from three independent experiments. Significant differences were detected by repeated-measure anova (*P*< 0.0001), and both RrgA and RrgC binding was significantly different from RrgB and GFP binding at 50 and 100 μg ml^−1^ by *post hoc* Bonferroni analysis (*P < 0.001). D–G. Pre-incubation of A549 cells with purified RrgA protein inhibits pilus-mediated adherence. A549 cells were pre-incubated with media alone (D), media containing 100 μg ml^−1^ of RrgA (E), or 100 μg ml^−1^ of GFP (F). After pre-incubation, A549 monolayers were infected with *S. pneumoniae* strain T4. Cells were stained with phalloidin (red) anti-*S. pneumoniae* capsule antibody (green), and imaged with a confocal microscope. RrgA pre-incubation inhibits the adherence of strain T4 to the A549 cells (E versus D), while the negative control GFP protein does not (F versus D). Scale bar is 20 μm. Adherent bacteria were counted, and the number of bacteria adherent to 100 A549 cells is shown in G (*n* = 6 fields, *P= 0.0002, **P= 0.8).

As *rrgA* expression is required for wild-type pilus-mediated adherence to A549 cell monolayers, we sought to characterize the binding of isolated His-tagged RrgA protein. Adherent A549 cell monolayers were incubated with RrgA in media in the absence of serum, the cells were washed, and the protein was subsequently detected immunologically. Abundant RrgA was detected on the surface of monolayers, suggesting that the recombinant subunit had bound to the adherent cells (Fig. 2A1). Further, it appeared that RrgA binding occurred in small patches or microdomains on the monolayer surface. Fluorescence was not due to binding of the anti-RrgA antibody to the A549 cell surface, as exclusion of the RrgA protein abolished fluorescent signal (Fig. 2A3), although cells were present (Fig. 2A2 and A4). Green fluorescence protein (GFP) was used as a negative control, and did not bind to A549 monolayers at an equivalent dose used for RrgA (Fig. 2B1).

To directly compare the capacity of the three *rlrA* pilus islet structural gene products to bind to target respiratory epithelial cells, His-tagged recombinant RrgA, RrgB and RrgC were cloned from the pathogenic TIGR4 strain of *S. pneumoniae* and produced in *Escherichia coli* and purified. Binding of purified pilus subunits to A549 cells in suspension was evaluated by antibody detection and FACS analysis at a range of protein doses. Increasing doses of recombinant RrgA resulted in a significant increase in the mean fluorescence intensity by FACS analysis when compared with GFP binding, and as little as 5 μg ml^−1^ of RrgA resulted in a detectable increase (Fig. 2C). Addition of RrgC at identical concentrations also increased the fluorescence, although not as significant as those observed with RrgA (Fig. 2C). Addition of RrgB did not significantly alter the fluorescence of target A549 cells. These studies suggest that RrgA has the highest affinity for A549 cells and binds at μg ml^−1^ concentrations, although RrgC may also have relevant adhesive properties.

To prove that the capacity of recombinant RrgA to bind to A549 cells is related to *rrgA*-mediated adherence of piliated pneumococci, A549 monolayers were pre-incubated with recombinant RrgA protein before addition of wild-type pneumococci, resulting in fewer adhering pneumococci than observed in the absence of pre-incubated RrgA (compare Fig. 2E with 2D). To control for increases in adherence from non-specific protein binding to epithelial cells, A549 cells were pre-incubated with GFP protein at identical concentrations, which had no effect on adherence (Fig. 2F). These data were quantified and found to be statistically significant (Fig. 2G), supporting a role for RrgA in pilus-mediated adherence.

### RrgA is found in monomeric form on the cell wall in the absence of other pilus subunits

**Fig. 3:**
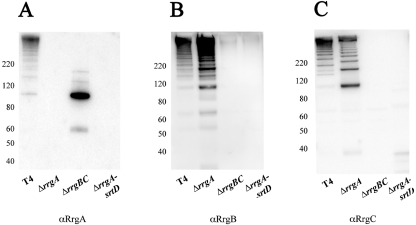
The pneumococcal pilus is expressed in the absence of RrgA. Production of polymeric high-molecular-weight extracellular pili in isogenic sets of pneumococci was evaluated by preparation of cell wall-associated proteins and immunoblotting for pilus subunits RrgA (A), RrgB (B) and RrgC (C). In all cases, cell wall proteins were separated by 4–12% gradient SDS-PAGE, electrotransferred to a PVDF membrane, and probed for pilus subunits. Approximate molecular weight in kDa, based on marker proteins, are indicated on left. A. Immunoblotting for RrgA in wild-type T4, T4Δ*rrgA* (‘Δ*rrgA’*), T4Δ*rrgBC* (‘Δ*rrgBC’*), and T4Δ(*rrgA-srtD*) (‘*rrgA-srtD*’). B. Immunoblotting for RrgB in wild-type piliated T4, T4Δ*rrgA*, T4Δ*rrgBC* and T4Δ(*rrgA-srtD*), labelled as in A. C. Immunoblotting for RrgC in wild-type piliated T4, T4Δ*rrgA*, T4Δ*rrgBC*, and T4Δ(*rrgA-srtD*), labelled as in A. These data demonstrate that extracellular RrgB-positive, RrgC-positive pili are formed in the absence of *rrgA* expression, but not in the absence of expression of *rrgB* and *rrgC*. In the absence of RrgB and RrgC, RrgA is found as a 100 kDa monomer.

Given that RrgA is required for pilus-mediated adherence, we sought to determine the form of RrgA on the bacterial surface in the presence and absence of RrgB and RrgC expression. Cell wall-associated material was prepared by digestion with the muramidase mutanolysin, and separated by SDS-PAGE for Western blot analysis. In wild-type organisms, RrgA was incorporated into high-molecular-weight ladders consistent with pilus production, as well as in a 100 kDa monomer (Fig. 3A, lane 1). No RrgA was detected in material derived from the T4Δ*rrgA* (Fig. 3A, lane 2) and the T4Δ*rrgA-srtD* negative control (Fig. 3A, lane 4). In organisms that lack both of the other two structural subunits of the pilus, RrgB and RrgC, RrgA was found predominantly in the 100 kDa monomeric form (Fig. 3A, lane 3).

Both RrgB and RrgC were found in high-molecular-weight ladder polymers in the presence (Fig. 3B and C, lane 1) and absence of RrgA (Fig. 3B and C, lane 2). These observations demonstrated that mutation of *rrgA* did not abolish pilus assembly, and that RrgA was found in a cell wall-associated monomer in the absence of pilus expression. It is notable that the banding pattern and intensity of RrgB and RrgC staining of pilus polymers was different in wild-type and Δ*rrgA* strains.

**Fig. 4:**
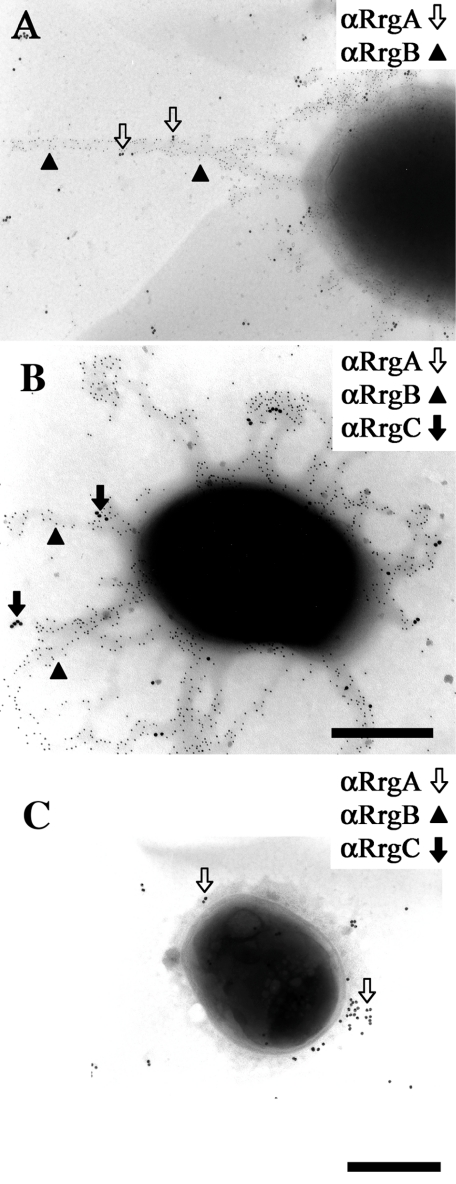
The pneumococcal pilus is observed in the absence of RrgA expression. A. Immunogold detection of RrgA (10 nm gold particles, indicated by open arrow) and RrgB (5 nm gold particle, indicated by arrowheads) in wild-type T4. B. Immunogold detection of RrgA (10 nm gold particles, open arrow), RrgB (5 nm gold particles, arrowhead) and RrgC (15 nm gold particles, closed arrow) in T4Δ*rrgA.* Scale bar is 200 nm. No RrgA was detected in T4Δ*rrgA* preparations. Note that polymeric RrgB- and RrgC-positive pili are present in T4 strains in the presence (A) or absence (B) of *rrgA* expression. C. Immunogold detection of RrgA (10 nm gold particle, open arrow), RrgB (5 nm gold particles, arrowhead), and RrgC (15 nm gold particles, closed arrow), in T4Δ*rrgBC.* Scale bar is 200 nm. No RrgB or RrgC was detected in T4Δ*rrgBC* preparations. Note that RrgA is found in extracellular pili in wild-type T4, but in association with the cell wall in T4Δ*rrgBC*, where no pili are observed.

We sought to verify the results of the Western blot analysis by immunoelectron microscopy. RrgA and RrgB were localized on the surface of wild-type piliated bacteria with specific antisera. Abundant RrgB was detected throughout the length of extracellular pili in wild-type organisms (Fig. 4A). As previously reported, RrgA was found periodically throughout the length (LeMieux *et al*., 2006) and at the base of wild-type pili (Barocchi *et al*., 2006) (Fig. 4A). RrgC was less abundant and could be found alone or in association with RrgA (F. Hilleringmann and I. Ferlenghi pers. comm.). The distribution of both RrgB and RrgC in extracellular pilus fibres of an *rrgA* mutant was indistinguishable from wild-type samples (Fig. 4B and data not shown), supporting the hypothesis that disruption of RrgA expression does not abrogate pilus formation. In Δ*rrgBC* bacteria, RrgA was detected exclusively in association with the pneumococcal cell surface, and with a higher frequency than when found in association with the pilus polymer (Fig. 4C). These data support the conclusion drawn from Western analysis, that expression of RrgA is not required for the formation of polymeric extracellular pili, and moreover, that monomeric RrgA is cell wall-associated in Δ*rrgBC* organisms and, at such a location, is capable of interacting with host cells.

### Expression of the *rrgA* gene is required for pilus-mediated pathogenicity

**Fig. 5:**
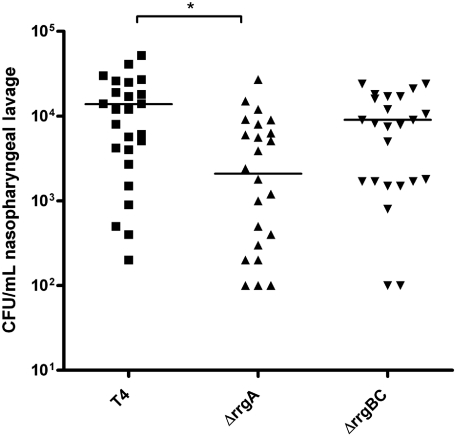
RrgA is involved in pilus-mediated colonization of the upper respiratory tract in mice. Mice were challenged intranasally with a low dose of T4, T4Δ*rrgA* or T4Δ*rrgBC* (*c*. 7 × 10^4^ cfu per mouse). Bacterial density in the nasopharynx was determined 7 days post infection. Mice infected with T4Δ*rrgA* had significantly fewer bacteria in the nasopharynx than mice infected with T4 or T4Δ*rrgBC* (*P< 0.01, *n* = 3 independent replicates of 10 mice per group per replicate, using the Kruskal–Wallis test with Dunn's post testing).

In addition to adherence to host epithelium, failure to express RrgA in a mouse model of pneumonia significantly impairs bacterial competition (Hava and Camilli, 2002), supporting an important role for this gene product *in vivo* in the respiratory tract. It has also been reported that the pneumococcal pilus confers a more pathogenic phenotype in a mouse model of intraperitoneal sepsis (Barocchi *et al*., 2006), although no specific role for any single subunit has been demonstrated. Given the importance of RrgA in adherence *in vitro*, we tested the contribution of RrgA in a mouse model of colonization of the upper airways. Mice were challenged with a low dose of piliated T4, T4Δ*rrgA* or T4Δ*rrgBC* (*c*. 7 × 10^4^ cfu per mouse). The bacterial density in the nasopharynx of mice infected with the strain lacking only RrgA was significantly lower compared with wild-type T4 1 week post infection (*P*< 0.01) (Fig. 5). Mice infected with the strain lacking both RrgB and RrgC, and therefore the pilus polymer, did not exhibit any significant difference in upper respiratory bacterial density when compared with wild-type bacteria. These data support a critical role for RrgA in pilus-mediated colonization in a host animal.

## Discussion

This study demonstrates an important role for one of three structural subunits, RrgA, in function of a polymeric extracellular Gram-positive pilus. This conclusion is based on genetic manipulation of bacterial gene expression in association with studies of host cells *in vitro* and mucosal surfaces *in vivo*, as the binding activity of recombinant protein *in vitro*.

Specifically, adherence of the piliated Δ*rrgA* mutant demonstrated adherence levels comparable to non-piliated organisms. Failure to express the other two subunits did not significantly reduce adherence below wild-type levels. These data prove that RrgA is an adhesin, and are verified genetically by complementation and gene dosage overexpression, and biochemically by showing that recombinant RrgA binds to A549 cells. As noted, studies of the group B streptococcus, *S. agalactiae*, indicate that the homologue of *rrgA* is required for pilus-mediated adherence to A549 human respiratory epithelial cells (Barocchi *et al*., 2006; Dramsi *et al*., 2006) and to human brain microvascular endothelial cells (Maisey *et al*., 2007), and the *rrgA* and *rrgC* homologues of the group A streptococcus, *S. pyogenes* (Abbot *et al*., 2007) and *C. diphtheriae* (Mandlik *et al*., 2007) play critical roles in adherence of these organisms to host cells. These studies provided genetic and biochemical evidence, demonstrating that mutants in *rrgA* homologues were less adherent than wild-type piliated organisms, and Maisey and colleagues (2007) showed that heterologous expression of the *S. agalactiae* homologue of *rrgA*, *pilA,* in *Lactococcus lactis* enhances adherence. Proof that ancillary subunits, not necessary for pilus polymerization, are essential in pilus-mediated adherence is therefore emerging in multiple systems, as shown 20 years ago for uropathogenic *E. coli* P-pili (Lindberg *et al*., 1987; Lund *et al*., 1987).

All three pneumococcal pilus subunits have MSCRAMM motifs (Hava and Camilli, 2002), suggesting possible roles in adherence to host structures (Patti *et al*., 1994). However, RrgA, the largest of the three pneumococcal pilus subunits, contains additional vWA (Colombatti and Bonaldo, 1991) and RGD domains (Ruoslahti and Pierschbacher, 1986), both of which have been associated with adhesion to extracellular proteins, and both of which are lacking in the two other pilus proteins. Additional experiments are required to elucidate whether these domains are involved in RrgA-mediated adherence and pathogenicity. The importance of RrgA and the relatively low sequence variation in RrgA among different piliated pneumococcal strains (B. Henriques Normark *et al*., unpubl. obs) makes RrgA an attractive vaccine candidate (Gianfaldoni *et al*., 2007).

It is not clear why purified recombinant RrgC is capable of binding to A549 cells in suspension, like RrgA, despite lack of genetic support for the role of this gene product in adherence of live bacteria. As noted, the *rrgC* homologue of the *S. pyogenes* pilus (Abbot *et al*., 2007) and the *C. diphtheriae* pilus (Mandlik *et al*., 2007) has been shown to play an important role in adherence to tissues and cells. It is possible that RrgC does play an adhesive role *in vivo* during colonization or infection of a human host, and our *in vitro* adherence model fails to recapitulate appropriate conditions. Indeed, an ELISA-based assay provided evidence that RrgA was able to bind a panel of extracellular matrix proteins *in vitro* (M. Hilleringmann, pers. comm.), which have been shown to serve as receptors for other MSCRAMM proteins (Patti *et al*., 1994). In addition, it is possible that A549 cells express low levels of the RrgC receptor, preventing detectable changes in bacterial binding to cells, but permitting changes in sensitive FACS-based assays using purified proteins. Regardless, neither *rrgA* nor *rrgC* is necessary for the formation of extracellular pili (this report; A. Nelson and B. Henriques Normark, in preparation; LeMieux *et al*., 2006). Thus, RrgA and RrgC may be attached along the pilus, facilitating recognition of and binding to target receptors. In addition, it is likely that presentation of adhesive proteins on long pili that extend beyond charged surface molecules, such as the capsule and the cell wall, may overcome a ‘hiding effect’ to which other adhesive components are subject.

Upper respiratory tract colonization in an animal model of colonization is significantly affected by *rrgA* sufficiency, supporting the important role that RrgA plays in mediated host–bacterial interactions. This observation is in concordance with previous reports identifying *rrgA* and the *rlrA* islet-associated sortase, *srtB*, as ‘essential’ for colonization of the upper respiratory tract, and *rrgA* and another *rlrA* islet-associated sortase, *srtD*, as ‘essential’ for infection of the lower respiratory tract of mice (Hava and Camilli, 2002). Notably, however, this study failed to detect a defect in *rrgA*, *srtB* or *srtD* in a mouse model of sepsis. Regardless of specific differences, these studies and others (Barocchi *et al*., 2006) support the pneumococcal pilus as a virulence factor, and the present work demonstrates that RrgA is an important component.

As in the adherence model, the *rrgBC* mutant was found to be no different from wild-type organisms, suggesting that only *rrgA* is responsible for the increased colonization of piliated organisms. Δ*rrgB* and Δ*rrgBC* strains lack high-molecular-weight extracellular pilus polymers, and in such strains RrgA seems to be confined to the cell wall. Surprisingly, these data suggest that RrgA alone is capable of increasing both adherence and pathogenicity, even in the absence of the two other pilus subunits. A similar situation, however, also prevails in the previously mentioned uropathogenic *E. coli* P-pili, where the pilus-associated PapG adhesin mediates bacterial adherence to human cells, even in the absence of the major pilin (Uhlin *et al*., 1985). Importantly, however, the PapG adhesin is still anchored to the outer membrane by a short fibrillum provided by another minor pilus subunit (Kuehn *et al*., 1992), while RrgA is directly anchored to the cell wall. In the absence of pilus expression, RrgA is expected to be found ‘below’ the capsule, like other putative adhesive structures, including phosphorylchlorine (Cundell *et al*., 1995) and the choline-binding protein A (CbpA) gene product (Zhang *et al*., 2000), among others (Hammerschmidt, 2006). Indeed, this has been shown to be the case, using immunofluorescence detection of RrgA as proxy for surface exposure, where RrgA is only detectable in the absence of RrgB expression if capsule expression is also inactivated (A. Nelson, in preparation). Further, it has also been shown that different capsules permit greater or lesser access to such surface proteins (Abeyta *et al*., 2003), meaning that encapsulation is not incompatible with surface access.

This work raises questions as to the actual function of the pilus fibre itself, adhesive properties aside. Why manufacture a large and costly pilus on which to place an adhesin if the adhesin functions perfectly well *in vitro* and *in vivo* in the absence of such a fibre? It is possible that the assays employed to date, *in vitro* adherence of immortalized cells in culture and *in vivo* colonization of a non-cognate model host, do not properly test the functional benefit provided by a pilus fibre. It is also possible that the other proteins, RrgB and RrgC may provide additional functions. For example, the recent finding that pili of *Enterococcus fecalis* promote biofilm formation (Nallapareddy *et al*., 2006) suggests that Gram-positive pili not only function in adherence to eukaryotic cells, but also may promote bacterial–bacterial interactions. Finally, it is worth considering whether RrgA may have additional, non-adhesive functions that require a pilus for proper activity, interaction with host phagocytes, for example. Regardless of these hypotheses, we present support for at least one of the ancillary subunits of such a Gram-positive pilus as a *bona fide* adhesin, and note that future work will address the role of the pilus fibre upon which the adhesin is normally found.

## Experimental procedures

### Bacterial strains, media and growth conditions

Pneumococcal strains and isogenic mutant derivatives are described in Table 1.

**Table 1:** Characteristics of strains used in the study.

Strain	Relevant characteristics	Source/reference
T4	TIGR4 piliated invasive isolate	Barocchi *et al*. (2006)
T4Δ*rrgA*	Erm^R^, piliated strain lacking *rrgA*	This study
T4Δ*rrgBC*	Erm^R^, non-piliated strain lacking *rrgB* and *rrgC*	This study
T4Δ(*rrgA-srtD*)	Erm^R^, non-piliated strain lacking *rrgA, rrgB, rrgC, srtB, srtC* and *srtD*	Barocchi *et al*. (2006)
T4Δ*rrgA*∇(*lacE*::*rrgA*)	Erm^R^, Spect^R^, piliated strain lacking endogenous *rrgA*, transgenic second copy of *rrgA* in *lacE* locus	This study
T4∇(*lacE*::*rrgA*)	Spect^R^, piliated strain with transgenic second copy of *rrgA* in *lacE* locus	This study

The insertion–deletion mutagenesis used for most strains is described elsewhere (Barocchi *et al*., 2006; Lau *et al*., 2002). Briefly, approximately 1 kB of upstream and downstream fragments of the relevant open reading frame (ORF) was amplified by polymerase chain reaction (PCR), digested with ApaI and BamHI respectively, and ligated to either end of a double-digested erythromycin-resistance cassette (Erm^R^, GenBank accession AB057644). Upstream sequence of *rrgA* was amplified with primers RrgA-1 and RrgA-2, and downstream sequence with RrgA-3 and RrgA-4. Upstream sequence of *rrgB* was amplified with primers B-1 and RrgB-2, and downstream sequence of *rrgC* with RrgC-3 and RrgC-4. The erythromycin-resistance gene was amplified with primers Erm-5′ and Erm-3′. All primers are described in Table 2. The ligated knock-out construct was transformed (Bricker and Camilli, 1999) into the appropriate wild-type pneumococcal strain and plated on blood plates with erythromycin (1 μg ml^−1^). Mutants were checked by PCR, sequencing and immunogenicity, thereby also demonstrating a lack of polar effects of deletion of one subunit gene on expression of others.

**Table 2:** Primers used in the study, with restriction enzymes sites.

Primer name	Sequence
RrgA-1	CAAGGTCCAAACCTACTGAAC
RrgA-2	GCGGGCCCCTGAGATATACAGCACAGTCC
	ApaI site underlined
RrgA-3	CGGGATCCCCAGCGGGTTACGAGTTTAC
	BamHI site underlined
RrgA-4	CAACAAGGTGGAACACAGG
RrgB-1	GTGTAACAGGTCTGTACCTTG
RrgB-2	CGGGCCCGGTAACAGATGTTGTTGTCGTC
	ApaI site underlined
RrgC-3	CGGATCCGGTCTAGAGTATGGGACATAC
	BamHI site underlined
RrgC-4	GCAATACCTCTTCAGCAGTAC
Erm-5′	TTTTTGGGCCCTTCGTGTTCGTGCTGACTTGC
	ApaI site underlined
Erm-3′	TTTTTGGATCCGATGTTGCTGATTAAGACGAGC
	BamHI site underlined
NlacEF	GGGTATTGTGTGGATTAAAAAGG
NlacER	ACTGGTTTCTACAGGCTTGATTAG
SP0479R	GCTGTGTAGTAAGTTTTTCCA
SpcR1	CCCAGATCTCAATTTTTTTATAATTTTTTT
SP0479-RrgA	TGGAAAAACTTACTACACAGCTTACGGATGTTTCCGTGTGTA
RrgA-aad9	CAGATGAAAAAAATTATAAAAAAATTGAGATCTGGGTGCTACGTTTGTTAGTGAACG

Complementation *in trans* was performed using the methods of Iyer *et al*. (2005). Briefly, upstream (5′) and downstream (3′) portions of the *ccpA-trans*-complemention construct in the *lacE* locus (TIGR4 SP0474–SP0478) of strain AC1933 (Iyer *et al*., 2005) (generously donated by A. Camilli, Boston, MA) were amplified by PCR with primers NlacEF and SpcR1, and SP0479R and NlacER respectively. Separately, a promoterless 2.8 kb *rrgA* fragment was amplified from wild-type T4 with primers SP0479-RrgA and RrgA-aad9, both containing overhangs homologous to the distal parts of the *lacE* fragments. The 1.6 kb NlacEF/SpcR1 *lacE* upstream product was spliced to the SP0479-RrgA/RrgA-aad9 *rrgA* product by overhang extension with Phusion polymerase (Finnzymes). The resulting 4.4 kb fragment was amplified with primers NlacEF/SP0479-RrgA and spliced in a second overhang extension reaction to the 0.8 kb NlacER/SP0479R *lacE* downstream fragment. After amplification with primers NlacEF/NlacER, the resulting 5.2 kb product was gel-purified, checked by sequencing, and transformed into T4Δ*rrgA* and T4 wild-type to generate a *rrgA trans*-complemented strain, T4Δ*rrgA*∇(*lacE*::*rrgA*), and a RrgA-overproducing strain, T4∇(*lacE*::*rrgA*) respectively.

Unless otherwise noted, bacteria were streaked from frozen stocks onto blood plates with appropriate selection for overnight growth, inoculated briefly into pre-warmed dextrose–serum (DS) medium (OXOID Manual, 1990), and then DS was inoculated into pre-warmed C + Y medium to achieve an OD_620_ = 0.05. Cultures were permitted to grow to mid-log (OD_620_ = 0.5) before collection for experimentation. Bacterial medium was produced by the Karolinska Microbiology Laboratory.

### Cell culture techniques and adherence assays

A549 human respiratory epithelial cells (ATCC number CCL-185) were grown to confluency in polystyrene 24-well plates (Corning) on coverslips in RPMI 1640 medium supplemented with 10% heat-inactivated fetal calf serum and 2 mM glutamine. All cell culture reagents were purchased from Gibco/Invitrogen.

Before use, the monolayers were washed twice with PBS. *S. pneumoniae* cells grown to mid-log phase (OD_620_ = 0.5) in brain heart infusion broth were resuspended in cell culture medium without serum. Bacterial suspensions were applied to A549 cell monolayers with a multiplicity of infection (moi) of approximately 100, centrifuged at 1000 r.p.m. for 2 min at 4°C, and subsequently incubated for 30 min at 37°C, 5% CO_2_/95% air atmosphere. After incubation, the infected monolayers were washed three times with PBS to remove non-adherent bacteria, fixed in 3% paraformaldehyde for 15 min, washed three times and treated with NH_4_Cl (10 mM) overnight. For fluorescence microscopy, the coverslips were washed two times with PBS with 0.1% saponin and labelLed for 30 min with anti-serotype 4 typing serum (Statens Serum Institute, Denmark) in PBS with 10% horse serum and 0.1% saponin. After washing two times, bacteria were visualized with Cy2-labelled donkey anti-rabbit antibodies (Jackson ImmunoResearch), and F-Actin with rhodamine-conjugated phalloidin (Molecular Probes). Cy2-labelled bacteria per 100 epithelial cells were counted on by fluorescence microscopy, each determination performed in triplicate, and three independent determinations were made.

### Production of purified proteins and binding to epithelial cells

Recombinant His-tagged pneumococcal pilus subunit proteins, RrgA, RrgB and RrgC, were produced as previously described (Barocchi *et al*., 2006). Briefly, the ORFs were cloned from TIGR4 chromosomal DNA by PCR, digested appropriately, and ligated into the *E. coli* expression plasmid, pET-21b+ (Invitrogen). The expression vector was transformed into *E. coli* BL21 star (DE3), induced with IPTG, and purified on a nickel column, as per the manufacturer's instructions (Invitrogen).

A549 were non-enzymatically detached from the support by using cell dissociation solution (Sigma), harvested and resuspended in Dulbecco's modified Eagle's medium (DMEM) supplemented with 1% BSA, in the absence of serum and antibiotics. The cells were mixed with either medium alone or three concentrations (5, 50 and 100 μg ml^−1^) of pilus subunits RrgA, RrgB and RrgC or GFP suspended in media and incubated for 2 h on ice. After two washes with 1% BSA in PBS, cells were incubated with antibodies against each protein for 1 h on ice. After two additional washes, the preparations were incubated with 488 Alexa Fluor secondary antibodies (Molecular Probes), and 10 000 cells were analysed with a FACS-Scan flow cytometer. Antibody cross-reactivity was negative by immunofluorescence and FACS analysis (data not shown).

For binding assay on adherent A549 respiratory epithelial cells, RrgA protein was suspended in media in the absence of serum and antibiotics at 100 μg ml^−1^, and incubated on cell monolayers grown on 13 mm glass coverslips for 2 h on ice. Negative control wells were treated and analysed in parallel with media in the absence of any exogenous protein. Subsequently, cells were washed three times with PBS, and fixed with 2.5% paraformaldehyde. Cells were then incubated with antibodies against each protein for 1 h at room temperature. After two additional washes, the preparations were incubated with 488 Alexa Fluor secondary antibodies and phalloidin (Molecular Probes).

For protein-mediated inhibition of bacterial adherence, 100 μg ml^−1^ of RrgA or GFP was incubated in DMEM plus 0.1% BSA for 30 min, with A549 cells on coverslips in a CO_2_ incubator. After three washes with media, the cells were incubated with T4 (100 moi) for 30 min in a CO_2_ incubator. After three washes with media, coverslips were fixed and stained with phalloidin (red) and with rabbit polyclonal omnisera against all known pneumococcal serotypes (Staten Serum Institute). Cells were imaged with a confocal microscope, and six different microscopic fields were counted.

### Cell wall preparations and immunoblotting

Cell wall-associated proteins were isolated from genetically defined strains of *S. pneumoniae* as previously described (Barocchi *et al*., 2006). Briefly, 5 ml mid-log culture was resuspended in 500 μl of 50 mM Tris-HCl, pH 6.8, with 125 U of mutanolysin (Sigma) and incubated for 2 h at 37°C with rotation. After three cycles of freezing and thawing, cellular debris was removed by centrifugation at 13 000 r.p.m. for 15 min, and protease inhibitor was added, as per the manufacturer's instructions (Roche complete protease inhibitor cocktail). Total protein was quantified by a modified Bradford test (Bio-Rad), and equal amounts of protein were mixed with NuPage sample buffer and β-mercaptoethanol, boiled at 100°C for 10 min, and loaded onto 4–12% NuPage Tris gels (Invitrogen). The gel was run at 4°C for approximately 4 h to isolate high-molecular-weight species, and electrotransferred to PVDF membranes (Invitrogen). The membrane was then immunoblotted for pneumococcal pilus-associated antigens using mouse anti-RrgA, rabbit anti-RrgB, or rabbit anti-RrgC antisera, previously described (Barocchi *et al*., 2006) or generated by identical approaches.

### Immunoelectron microscopy

Immunogold electron microscopy of pilus expression in TIGR4 was performed as previously described (Barocchi *et al*., 2006). Briefly, bacteria were grown overnight in THY medium, diluted and permitted to re-expand to OD_620_ = 0.5 before centrifugation and resuspension in PBS. Resuspended bacteria were spread onto Formvar-coated nicket grids to air-dry, and subsequently formaldehyde-fixed before staining with 1:10 dilutions of monoclonal anti-RrgB, anti-RrgA or anti-RrgC in 1% normal rabbit serum, 1% BSA, in PBS. Samples were washed before secondary goat anti-mouse IgG conjugated to gold particles was added at 1:20 dilution. Samples were then washed, fixed again, and stained with 1% uranyl acetate, before analysis in a Philips CM10 transmission electron microscope.

### Animal challenge

The intranasal pneumonia model was performed as previously described (Albiger *et al*., 2005). T4 and its respective isogenic mutants were grown to logarithmic phase (OD_620_ = 0.5). The mice were inoculated intranasally with ∼7 × 10^4^ cfu. Seven days post infection, viable bacteria in the nasopharynx were quantified by flushing 200 μl PBS through the trachea and collecting the fluid at the nostrils. Serial dilutions and plating on blood agar plates was used to determine bacterial density in nasopharyngeal lavage. The experiments were repeated over three independent replicates.

Six- to eight-week-old wild-type C57BL/6 were used for the intranasal bacterial challenges. The mice were age- and sex-matched, and kept under specific pathogen-free conditions. There were five animals per cage in standardized light/dark cycles, and they received food and water *ad libitum*. The health status of experimental animals was monitored according to the following scores: 0 = healthy; 1 = piloerection; 2 = reduced motility; 3 = fever, more pronounced reduced motility; 4 = 1,2,3 more pronounced; and 5 = moribund. Mice were sacrificed when they reached a score of ≥ 3. All animal experiments were conducted in conformity with the European Communities Council Directive 86/609/EEC and the Swedish Animal Protection Legislation.

### Statistical analyses

For adherence assays, data were analysed in GraphPad Prizm 4.0 (GraphPad Software), using a repeated-measure anova with *post hoc* Bonferroni testing. Data of cfu in the nasopharynx were analysed using non-parametric Kruskal–Wallis test with Dunn's post testing.

## Supplementary material

This material is available as part of the online article from: http://www.blackwell-synergy.com/doi/abs/10.1111/j.1365-2958.2007.05908.x (This link will take you to the article abstract).
