# Supplementary material for: RrgA is a pilus-associated adhesin in Streptococcus pneumoniae
Source: Mol Microbiol. 2007 Oct;66(2):329–40. doi: 10.1111/j.1365-2958.2007.05908.x (PMC2170534; doi:10.1111/j.1365-2958.2007.05908.x)
Supplement: Supplementary file 1 [file mmi0066-0329-SD1.pdf]

Figure S1

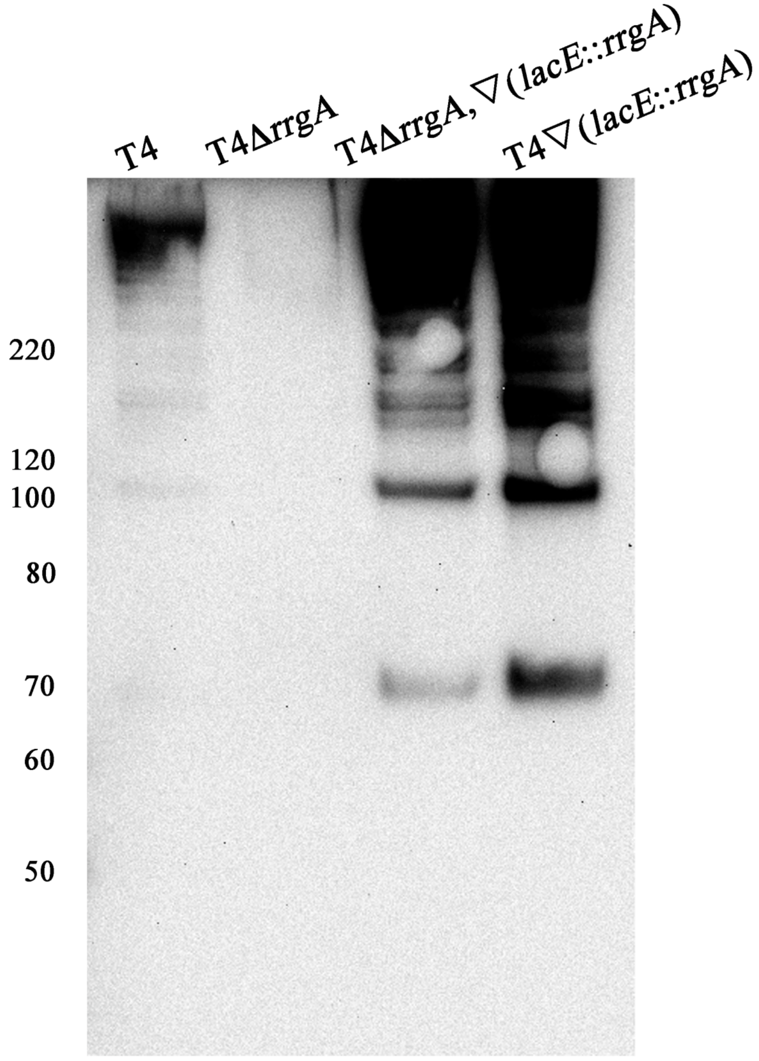

Figure S1. RrgA expression in *trans*-complemented and over-expressing strains. RrgA-positive pili were detected in cell wall-associated material from wild-type T4, T4Δ*rrgA*, T4Δ*rrgA*∇(*lacE::rrgA*), and T4∇(*lacE::rrgA*) by Western blotting, as shown in Fig 3. Note that RrgA-positive pili are not detected in T4Δ*rrgA* (lane 2), as shown in Fig 3. In contrast, pili are detected in wild-type T4 (lane 1), the *trans*-complemented strain T4Δ*rrgA*∇(*lacE::rrgA*) (lane 3), and T4∇(*lacE::rrgA*) (lane 4), which possesses two copies of *rrgA* on separate locations in the chromosome.
